# Puerarin Inhibits oxLDL-Induced Macrophage Activation and Foam Cell Formation in Human THP1 Macrophage

**DOI:** 10.1155/2015/403616

**Published:** 2015-10-21

**Authors:** Heng Zhang, Zhenhua Zhai, Hongyu Zhou, Yao Li, Xiaojie Li, Yuhan Lin, Weihong Li, Yueping Shi, Ming-Sheng Zhou

**Affiliations:** ^1^Department of Physiology, Liaoning Medical University, Jinzhou, Liaoning 121001, China; ^2^Department of Oncology, Cancer Center, Turmounesis and Microenvironment Laboratory, The First Affiliated Hospital, Liaoning Medical University, Jinzhou, Liaoning 121001, China; ^3^Vagelos Scholars Program of the Molecular Life Sciences, University of Pennsylvania, Philadelphia, PA 19104, USA; ^4^Department of Chinese Medicine, The First Affiliated Hospital, Liaoning Medical University, Jinzhou, Liaoning 121001, China

## Abstract

Puerarin, an isoflavone derived from Kudzu roots, has been widely used for treatment of cardiovascular and cerebral vascular diseases in China and other Asian countries. However, the underlying mechanisms are largely unknown. The present study investigated whether puerarin inhibited atherogenic lipid oxLDL-mediated macrophage activation and foam cell formation in human THP1 macrophage. Treatment with oxLDL significantly increased the mRNA expression of proinflammatory cytokines tumor necrosis factor *α* (TNF*α*, 160%) and interleukin (IL) 1*β* (13 fold) accompanied by upregulation of toll-like receptor 4 (TLR4, 165%) and the ratio of phospho-I*κ*B*α*/I*κ*B*α* in THP1 macrophage. Puerarin dose-dependently prevented an increase in oxLDL-induced proinflammatory gene expression with downregulation of TLR4 and the ratio of phospho-I*κ*B*α*/I*κ*B*α*. Furthermore, puerarin prevented oxLDL-mediated lipid deposition and foam cell formation associated with downregulation of scavenger receptor CD36. Flow cytometry analysis showed that puerarin reduced the number of early apoptotic cells of macrophages induced by oxLDL. Our results show that puerarin has anti-inflammatory and antiatherogenic effects in vitro; the underlying mechanisms may involve the inhibition of TLR4/NF*κ*B pathway and downregulation of CD36 expression. The results from the present study provide scientific evidence and may expand our armamentarium to use puerarin for prevention and treatment of cardiovascular and atherosclerotic diseases.

## 1. Introduction

Atherosclerosis, the primary cause of heart disease and stroke, is responsible for approximately 50% of all deaths in Western societies and is the leading cause of deaths worldwide [1]. Over the past decades, therapeutic options to treat atherosclerosis have been significantly improved [2, 3]. However, there is still an enormous unmet need: after 3 years, 20% of patients with acute coronary syndrome suffer from recurrent myocardial infarction despite optional medial therapy. Thus novel therapies to prevent atherogenesis or treat atherosclerosis are urgently needed [4]. Natural products, such as Chinese herbal medicine, could be an ideal source to develop safe and effective agents for treatment of cardiovascular and atherosclerotic diseases [5].

Atherosclerosis is considered as a chronic inflammatory disease of the arterial wall characterized with inflammation, oxidative stress, and immune system activation [4]. Monocytes/macrophages play a central role in atherosclerosis through the accumulation of cholesterol and production of inflammatory mediators and cytokines [6, 7]. Early atherogenesis is characterized by the adherence of blood circulating monocytes to vascular endothelium, then by their migration to the subendothelial space, and further by activation into macrophages [8]. Macrophages via their scavenger receptors take up oxidized LDL (oxLDL,) and other lipids, undergo activation, and produce cytokines, matrix metalloproteinases, and reactive oxygen species (ROS) while continuing to accumulate lipids and differentiate into foam cells to form the early lesions that mature into atherosclerotic plaques [9].

Puerarin (daidzein 8-C-glucoside, C_21_H_20_C_9_) is a major isoflavonoid compound isolated from the Chinese herb Kudzu roots, which is known as Gegen, one of the most popular Chinese traditional medicine [10]. Puerarin is available in common foods and has a long history for the treatment of cardiovascular and cerebrovascular diseases, including coronary artery disease, heart failure, hypertension, and myocardial infarction [10, 11], in China and other Asian countries. It has been proposed that puerarin may exert beneficial effects on cardiovascular system, including vasodilation, antioxidant, anti-inflammation, and antiplatelet aggregation [12–14]. Recent studies suggest that puerarin may have antiatherogenic effects [15, 16]. However, the underlying mechanisms remain largely unknown. The activated macrophages release of inflammatory cytokines and uptake of oxLDL forming foam cells are critical for the development of atherosclerotic plaque [17, 18]. It is well known that the oxidative modified LDLs such as oxLDL have atherogenic effects [9]. The present study investigated whether puerarin inhibited atherogenic lipid oxLDL-induced macrophage activation and foam cell formation, two key steps for development of atherosclerosis, in human THP1 macrophages, and elucidated the potential mechanisms of puerarin's antiatherosclerosis.

## 2. Methods

### 2.1. Materials

Human THP1 monocytes and supplemental medium were purchased from ATCC (ATCC TIB 202, Manassas, VA). Rabbit polyclonal anti-phospho-I*κ*B*α*, I*κ*B*α*, and CD36 antibodies were obtained from Santa Cruz Biotechnology (Santa Cruz, CA). Puerarin (98.9% purity) was purchased from Sigma Inco. (St Louis). oxLDL was purchased from Shanghai Luwen Biotech Inc. (Shanghai, China) and was used within 6 weeks after it was made. All other chemicals were of the best grade available from commercial sources.

### 2.2. Cell Culture

Human THP1 monocytes were cultured in RPMI 1640 medium supplemented with 10% fetal bovine serum (FBS), 10 mmol/L HEPES, 2 *μ*mol/L L-glutamine, 1 *μ*mol/L sodium pyruvate, 100 U/mL penicillin, 100 *μ*g/mL streptomycin, and 0.05 mmol/L 2-mercaptoethanol. The cells were cultured at 37°C, 95% humidity, and 5% CO_2_ and used between passages 4 and 16. The cells were seeded in six-well plates (5 × 10^5^ cells/well) and differentiated into macrophages by preincubation with 100 ng/mL phorbol 12-myristate 13-acetate (PMA, Sigma) for 36 hours. The cells were starved in serum-free RPMI 1640 medium for 24 hours before the experiments were performed. The cells were incubated with vehicle (equal amount of culture medium) and oxLDL (50 *μ*g/mL) with or without puerarin (from 10 *μ*g/mL to 100 *μ*g/mL) for 24 hours.

### 2.3. Real-Time PCR

The cells were harvested in 1 mL TRIzol reagent and total RNA (2 *μ*g) was reverse-transcribed using a superscript II RT first strand synthesis kit (Gibco, BRL) according to the manufacturer's instructions. Real-time PCR for tumor necrosis factor *α* (TNF*α*, assay ID: Hs00174128-m1) and interleukin 1*β* (IL1*β*, assay ID: Hs01336189-m1) was performed in 20 *μ*L reaction mixture containing an appropriately diluted (80 ng) cDNA solution, 0.1 *μ*mol/L of each primer, 0.2 *μ*mol/L probe, and PCR master mix assay kit (ABI) as previously described [19]. Relative quantities of each transcript were normalized by a housekeeping gene (GAPDH) and expressed as fold increase versus control. All primers were ordered through Life Technologies (ABI) online system with assay ID.

### 2.4. Western Blot Analyses

The cells were harvested with lysis buffer containing a protein inhibitor cocktail. Protein was quantified by Bio-Rad assay, and 30 *μ*g of total protein was first subjected to SDS-PAGE and then transferred to nitrocellulose membranes. The membranes were incubated with primary rabbit polyclonal anti-CD36, anti-toll-like receptor 4 (TLR4), anti-phospho-I*κ*B*α*, or anti-I*κ*B*α* (Santa Cruz Biotechnology) followed by incubation with a peroxidase-conjugated secondary antibody for 1 hour. Equivalence of protein loading and transfer was confirmed by reblotting the samples with anti-*β*-actin antibody (Santa Cruz Biotechnology). Immune reactive bands were detected by chemiluminescence and quantified by densitometry. Relative quantities of each protein were normalized by *β*-actin and expressed as fold increase versus control.

### 2.5. Foam Cell Formation

THP1 macrophages were preincubated with or without puerarin (10 to 100 *μ*g/mL) in chamber slides for 1 hour followed by incubation with oxLDL (50 *μ*g/mL) for 72 hours. Oil Red O staining was used to visualize lipid deposits and hematoxylin staining was used to show the nuclei as previously described [19]. The images were acquired using microscope Zeiss. Oil red staining intensity was measured by Image-Pro Plus and used for the quantitative analysis of the lipid accumulation in the foam cells.

### 2.6. Apoptosis Assay by Flow Cytometry Analysis

The cells were incubated with vehicle, oxLDL (100 *μ*g/mL), or oxLDL plus puerarin (100 *μ*g/mL) for 24 hours. Annexin V-FITC and PI staining were performed to detect early stage apoptosis in human THP1 macrophage followed by manufactory instruction. Briefly, the cells (1 × 10^5^) in 100 *μ*L of the binding buffer (10 mM HEPES pH 7.4, 140 mM NaCl, and 2.5 mM CaCl_2_) were mixed with 5 *μ*L of annexin V-FITC (BD Bioscience) and 10 *μ*L of PI (BD Bioscience). After 15-minute incubation at room temperature in the dark, the samples were immediately analyzed by flow cytometry. The relative number of cells that were annexin V-positive and PI-negative (early apoptotic cells) was determined.

### 2.7. Data Analysis

The results were expressed as mean ± standard error of the mean (SEM). Statistical analyses were performed by ANOVA with Bonferroni's correction for multiple comparisons. Significance was assumed at *P* < 0.05.

## 3. Results

### 3.1. Puerarin Prevented oxLDL Upregulation of CD36 Expression

**Figure 1:**
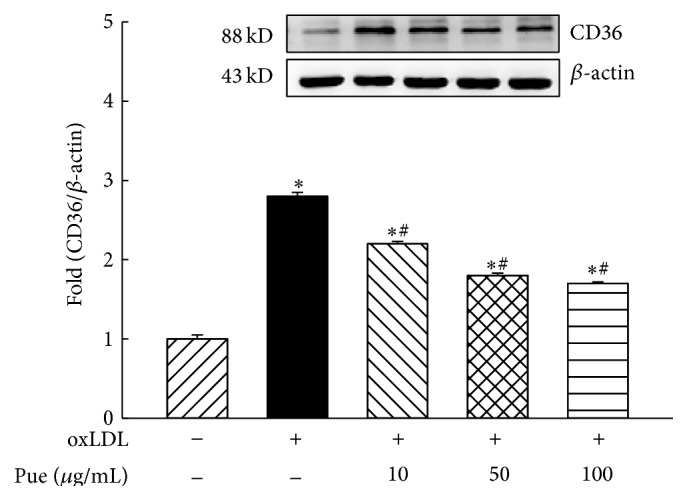
Effect of puerarin on the protein expression of CD36 in human THP1 macrophages. Incubation of oxLDL (50 *μ*g/mL) for 24 hours significantly increased the protein expression of CD36, and puerarin dose-dependently reduced CD36 expression. Pue: puerarin. Data is expressed as mean ± SEM. ^*∗*^ *P* < 0.05 versus control, ^#^ *P* < 0.05 versus oxLDL. *n* = 5 to 6.

CD36 is a member of the scavenger receptor class B family and a major scavenger receptor to uptake oxLDL (more than 70%) in human macrophage [20]. oxLDL is a major ligand of CD36 and plays an essential role in the pathogenesis of atherosclerotic plaque [9]. It has been shown that oxLDL upregulates CD36 expression in macrophages [20]. To investigate whether puerarin inhibits oxLDL upregulation of CD36 expression, we exposed THP1 macrophages to oxLDL (50 *μ*g/mL) with different doses of puerarin (10 *μ*g/mL to 100 *μ*g/mL) for 24 hours. As shown in Figure 1, oxLDL increased the protein expression of CD36 (170%), which was significantly inhibited by puerarin in a dose-dependent manner (*P* < 0.05).

### 3.2. Puerarin Inhibited Macrophage Activation in Response to Atherogenic Lipid oxLDL

**Figure 2:**
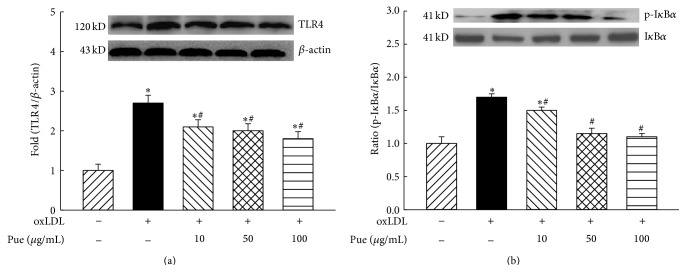
Effect of puerarin on the protein expression of toll-like receptor 4 (TLR4, (a)) and the ratio of phospho-(Ser32) I*κ*B*α*/I*κ*B*α* (b) in human THP1 macrophages. ^*∗*^ *P* < 0.05 versus control; ^#^ *P* < 0.05 versus oxLDL. *n* = 5 to 6.

The chronic stimulation of the innate immune system in response to endogenous ligands such as oxLDL is believed to be crucial to atherogenesis [21, 22]. Monocytes and macrophages have long been considered to be important immune effector cells that participate in host defense and the regulation of inflammation [8]. There are two major populations of macrophages based on their homeostatic activities and functions, inflammatory (M1) and tissue resident (M2) macrophages [23]. M1 macrophages are generated during cell-mediated immune responses [21, 23]. Activated M1 macrophages produce proinflammatory cytokines, including TNF*α,* IL1*β*, interferon *γ*, CXCL9, and inducible nitric oxide [23]. TNF*α* and IL1*β* are important M1 macrophage markers [23]. As shown in Figure 2, the mRNA expression of proinflammatory cytokines TNF*α* and IL1*β* was significantly increased in the cells treated with oxLDL. Puerarin prevented increase in the mRNA expression of these proinflammatory genes induced by oxLDL.

### 3.3. Puerarin Suppressed oxLDL Activation of TLR4/NF*κ*B Signaling

**Figure 3:**
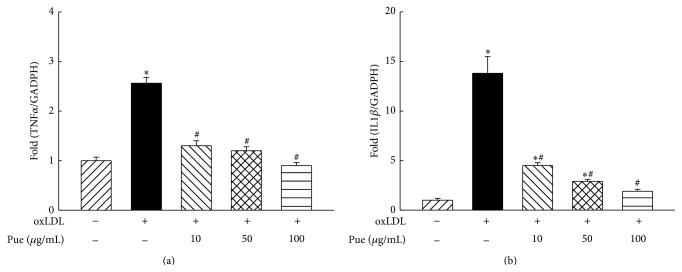
Effect of puerarin on the mRNA expression of tumor necrosis factor *α* (TNF*α*, (a)) and interleukin 1*β* (IL1*β*, (b)) in human THP1 macrophages. ^*∗*^ *P* < 0.05 versus control; ^#^ *P* < 0.05 versus oxLDL. *n* = 5 to 6.

It has been proposed that oxLDL induces inflammatory responses via activation of TLRs pathway [24]. As shown in Figure 3, oxLDL significantly increased the protein expression of TLR4, and puerarin dose-dependently suppressed oxLDL-induced TLR4 expression with maximal inhibition of 60% at 100 *μ*g/mL. NF*κ*B is a downstream molecule of TLR4 in the macrophage innate immune response [24, 25]. NF*κ*B activation is initiated from the phosphorylation of I*κ*B and inactivation of I*κ*B by phosphorylation and proteolysis leads to NF*κ*B translocation to the nucleus to trigger gene transcription. Increased phosphor (Ser32)-I*κ*B or the ratio of phospho-I*κ*B/I*κ*B is an index of NF*κ*B activation [25]. As shown in Figure 3, oxLDL treatment significantly increased the expression of phospho-I*κ*B*α* with a slight reduction in the expression of I*κ*B*α*; therefore, the ratio of phospho-I*κ*B*α*/I*κ*B*α* was increased in oxLDL-treated cells (*P* < 0.05). Puerarin dose-dependently reduced the expression of phospho-I*κ*B*α* and the ratio of phospho-I*κ*B*α*/I*κ*B*α* in oxLDL-treated cells (*P* < 0.05).

### 3.4. Puerarin Inhibited oxLDL-Induced Foam Cell Formation

**Figure 4:**
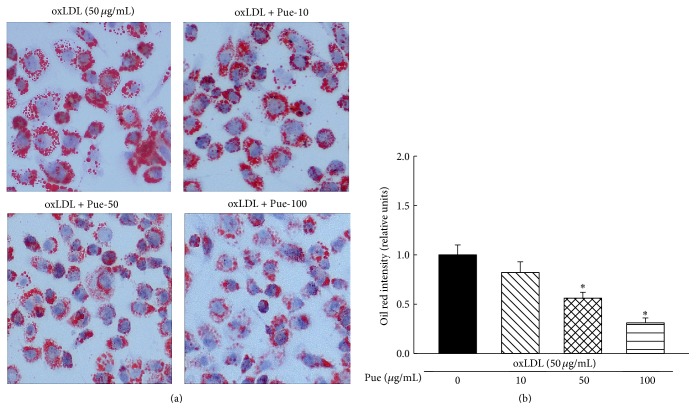
Effect of puerarin on oxLDL-induced foam cell formation in human THP1 macrophages. The THP1 macrophages were treated with oxLDL (50 *μ*g/mL) with or without puerarin at various doses (from 10 *μ*g/mL to 100 *μ*g/mL) for 72 hours. Oil red staining was performed, and oil red staining intensity was measured for the quantitation of the lipid accumulation in the foam cells. (a): representative images; (b): oil red intensity was quantified. ^*∗*^ *P* < 0.05 versus oxLDL. *n* = 5.

The macrophages uptake of oxLDL forming foam cell is a hallmark for atherosclerosis [18]. As shown in Figure 4, incubation of cells with oxLDL (50 *μ*g/mL) for 72 hours resulted in lipid deposition in macrophages and foam cell formation. The quantitative analysis of oil red staining intensity showed that puerarin significantly reduced oil red staining intensity in a dose-dependent manner, confirming that puerarin inhibits oxLDL-induced foam cell formation.

### 3.5. Puerarin Inhibited oxLDL-Induced Early Apoptosis of Macrophage

**Figure 5:**
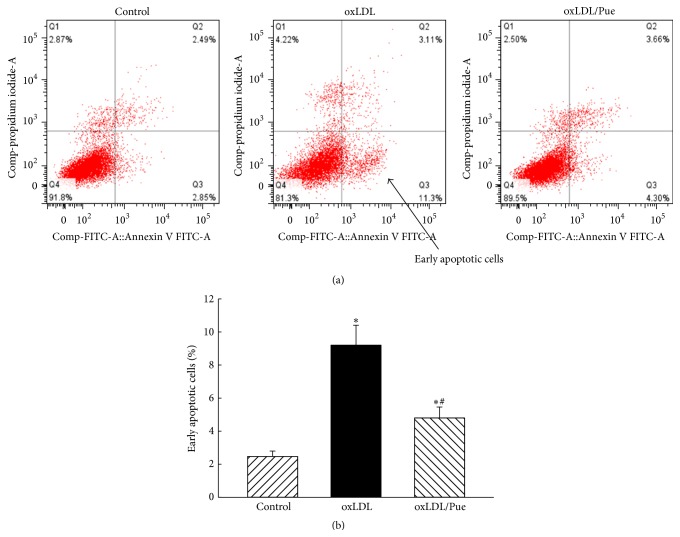
Effect of puerarin on oxLDL-induced early apoptotic cells in human THP1 macrophages. oxLDL (50 *μ*g/mL) significantly increased early apoptotic cells of macrophage (Annexin V-positive and PI-negative cells as indicated by arrow) determined by flow cytometry analysis, and treatment with puerarin (100 *μ*g/mL) significantly reduced oxLDL-induced apoptotic cells. (a): representative original trace of flow cytometry; (b): the quantitative analysis of early apoptotic cells of macrophages in bar graphs. *P* < 0.05 versus control; ^#^ *P* < 0.05 versus oxLDL. *n* = 6.

Macrophages in atherosclerotic lesions have been shown to have cellular characteristics of both necrosis and apoptosis [26]. The majority of apoptotic cells in atherosclerotic lesions are macrophages localized near the necrotic areas of advanced lesions [26]. As shown in Figure 5, oxLDL significantly increased the cell number that was annexin V-positive and PI-negative (early apoptotic cells, 9.2 ± 2.9% versus 2.47 ± 0.8% in control, *P* < 0.05). Puerarin reduced the number of early apoptotic cells in the cell treated with oxLDL (4.8 ± 1.6%, *P* < 0.05).

## 4. Discussion

Puerarin is a major isoflavonoid compound extracted from the Chinese medical herb Kudzu root [27, 28]. For more than 2000 years, Kudzu root has been used as a herbal medicine for the treatment of several diseases including fever, diabetes, and cardiovascular diseases in China and some Asian countries [10]. Currently, puerarin has been widely used for treatment of coronary artery disease and angina pectoris in Chinese traditional medical practice [10, 28]. A number of studies have shown that puerarin has cardiovascular protective effects such as antioxidant, vasorelaxation, and anti-inflammation and antiapoptosis in vascular endothelial cells [13, 14, 28, 29]. The present study demonstrated for the first time that in human THP1 macrophages puerarin dose-dependently suppressed atherogenic lipid oxLDL-induced macrophage activation and releases of inflammatory cytokines TNF*α* and IL1*β* associated with inhibition of innate immune pathway of TLR4/NF*κ*B, and puerarin inhibited oxLDL-mediated lipid deposition and foam cell formation with downregulation of CD36 expression and inhibited oxLDL-induced macrophage apoptosis. These data strongly suggest that puerarin has important anti-inflammatory antiatherosclerotic effects through modulation of the innate immune cells.

It has been well established that the elevation of plasma level of oxLDLs is associated with an increased risk for the development of atherogenesis. During atherosclerosis, heightened oxidative stress in the artery wall gives rise to oxidized forms of LDL that provoke an inflammatory response [30]. The macrophages recognize and take up these oxidized lipids through a family of pattern recognition receptors known as scavenger receptors. CD36 is the predominant scavenger receptor for oxLDL uptake in macrophages [20]. Interaction of CD36 with oxLDL on macrophages triggers a signaling cascade response that is both proinflammatory and proatherogenic [24]. CD36 is believed to play a critical role in the initiation of atherosclerotic lesions through its ability to bind and internalize modified LDL trapped in the artery wall, facilitating the formation of macrophage foam cells and the release of inflammatory cytokines from lipid-loaded macrophages in the arteries [9]. The present study demonstrated that oxLDL significantly increased the expression of CD36 in human THP1 macrophages, and puerarin dose-dependently inhibited oxLDL upregulation of CD36 expression accompanied by a reduction in foam cell formation. Since CD36 is critical for oxLDL uptake and foam cell formation in macrophage [9, 24, 31], other scavenger receptors such as scavenger receptor A and oxidized low-density lipoprotein receptor 1 (LOX1) may also participate in these processes mediated by oxLDL [24]. However, our previous study has shown that oxLDL did not affect the expression of scavenger receptor A and LOX1 in these cell lines and nicotine promoted foam cells and atherosclerosis via upregulation and stimulation of macrophage CD36 signaling [19]. Therefore, we speculate that inhibition of foam cell formation by puerarin may be at least in part attributed to its suppression of CD36 expression.

Inflammation plays a crucial role in the pathogenesis of atherosclerosis [4]. Inflammatory cytokines are uniformly more present in patients with atherosclerosis. Sites of atherosclerotic plaque development in the arterial wall are characterized by cholesterol accumulation and by peripheral blood monocytes and macrophage infiltration [22]. It is generally accepted that circulating inflammatory monocytes have a high capacity to migrate to tissues including the vascular wall, where they differentiate into inflammatory macrophages and foam cells in response to oxidized LDL [23]. The present study demonstrated that THP1 macrophages treated with oxLDL exhibited a significant increase in the mRNA expression of inflammatory cytokines TNF*α* and IL1*β*, two cytokine markers for inflammatory M1 macrophage; puerarin prevented an increase in oxLDL-induced macrophage release of the cytokines, suggesting that puerarin inhibits macrophage activation with anti-inflammatory effects.

It has been shown that oxLDL stimulates macrophage activation via the activation of TLRs/NF*κ*B immune pathway [24]. A recent elegant study demonstrated that oxLDL, after binding to CD36, facilitated heterodimer formation of TLR4 and TLR6 in macrophages, and this co-receptor complex activated downstream molecule redox-sensitive transcription factor NF*κ*B, consequently resulting in macrophage activation and release/production of inflammatory chemokines and cytokines [24]. Puerarin has been reported to inhibit TLR4 innate signaling pathway in cerebral ischemia/reperfusion-induced tissue [32]. Here we showed that puerarin inhibited oxLDL-induced macrophages release of inflammatory cytokines associated with reduction in expression of TLR4 and ratio of phosphor-I*κ*B*α*/I*κ*B*α*, suggesting that puerarin inhibits oxLDL-mediated macrophage activation via inhibition of TLR4/NF*κ*B pathway. In the unstimulated cells, NF*κ*B is present as an inactive, I*κ*B-bound complex in the cytoplasm. Many signals that lead to activation of NF*κ*B converge on the ROS-dependent activation of I*κ*B kinase (IKK) [33]. Activation of the IKK complex leads to the phosphorylation and degradation of I*κ*B, consequently activating NF*κ*B [25, 33]. Puerarin is an isoflavone with potent antioxidant effect and may inhibit NF*κ*B activation through suppression of ROS-dependent activation of IKK. In addition, our data showed that puerarin inhibited oxLDL-induced CD36 and TLR4 expression, and oxLDL can facilitate the interaction between CD36 and TLR4 to induce NF*κ*B activation [24]. Therefore, puerarin may also inhibit NF*κ*B activation through inhibition of interaction of CD36 with TLR4.

Macrophage apoptosis is a prominent feature of atherosclerotic plaque development. Macrophage apoptosis occurs throughout atherogenesis [26]. Increasing evidence suggests that advanced lesional macrophage apoptosis is associated with the development of vulnerable plaque [26, 34]. Vulnerable plaque increases risk to precipitate acute coronary syndromes like unstable angina and acute myocardial infarction. The present study showed that puerarin inhibited oxLDL-induced early apoptotic cells of macrophages, and the results may imply a potential therapy of puerarin for patients with unstable angina.

In summary, puerarin has a long history for the treatment of cardiovascular and vascular atherosclerotic diseases [10, 28]. The present study demonstrated that, in human THP1 macrophage, puerarin inhibited atherogenic oxLDL-induced macrophage activation and foam cell formation associated with downregulation of scavenger receptor CD36 expression and TLR4/NF*κ*B pathway, suggesting that puerarin has important anti-inflammatory and antiatherogenic properties. The cellular and molecular mechanisms of puerarin's antiatherosclerotic effects need to be further investigated. Our studies provide scientific evidence and may expand our armamentarium to use puerarin for prevention and treatment of cardiovascular and atherosclerotic diseases.
